# Inflammatory Factors Associated with Late-Life Depressive Symptoms in Oldest-Old Patients with Type 2 Diabetes

**DOI:** 10.3390/ijms27156975

**Published:** 2026-08-03

**Authors:** Malgorzata Gorska-Ciebiada, Maciej Ciebiada

**Affiliations:** 1 Department of Propaedeutics of Lifestyle Diseases, Medical University of Lodz, ul. Jaracza 63, 90-251 Lodz, Poland; 2 Department of General and Oncological Pulmonology, Medical University of Lodz, 90-549 Lodz, Poland

**Keywords:** IL-1β, IL-6, late-life depressive symptoms, oldest old, TNF-α, type 2 diabetes

## Abstract

While many studies indicate a bidirectional relationship between type 2 diabetes (T2DM) and depression, its precise nature remains unclear. The aim of the study was to determine the levels of inflammatory markers in oldest-old patients with diabetes and depressive symptoms and identify the risk factors associated with late-life depressive symptoms. In total, 195 participants with diabetes, aged 85 and above, were screened for depressive symptoms using the 30-item Geriatric Depression Scale (GDS-30). Clinical and biochemical data were also collected. Serum levels of inflammatory markers were measured via enzyme-linked immunosorbent assay (ELISA). Serum levels of TNF-α, IL-1β, and IL-6 were increased in individuals with depression and correlated with each other as well as with HbA_1c_ levels, GDS scores, FBG and BMI. The multivariable analysis confirmed that of the three studied biomarkers, only TNF-α is independently associated with depressive symptoms when adjusting for demographic factors (Model 1) and cardiovascular burden (Model 3); however, its significance disappeared when controlling for glycaemic parameters (Model 2). Only TNF-α is independently associated with depressive symptoms in oldest-old patients with T2DM, though this relationship is tightly dependent on glycaemic control. Future studies should clarify whether IL-1β and IL-6 contribute to depressive symptoms through distinct pathophysiological pathways.

## 1. Introduction

One of the most common chronic diseases worldwide is type 2 diabetes (T2DM). Its prevalence is expected to grow from 588.7 million in 2024 to 852.5 million by 2050 [1]. The condition primarily affects older adults, with 28.8% of people aged 65 and above in the United States being recognised as having diabetes [2]. It is known to be associated with a number of long-term complications that affect various organs, such as the cardiovascular system, eyes, kidneys, brain and nervous system. In addition, elderly patients often experience late-life depression and difficulties with cognitive function, which may have a bidirectional relationship with diabetes [3,4]: depression can increase the risk of developing diabetes due to behavioural changes, such as unhealthy diet or lack of physical activity, and conversely, the psychological burdens associated with managing a chronic disease can often trigger or exacerbate depressive episodes.

However, the nature of this relationship is not completely understood, although neurotransmitter imbalances and neuroendocrine dysregulation may play roles [5]. Chronic stress is known to induce hormonal disturbances, such as elevated cortisol levels and disrupted insulin and GLP-1 signalling, which in turn activate the hypothalamic–pituitary–adrenal axis (HPA) [5]. Persistent stimulation of the HPA, associated with both depression and diabetes, as well as obesity, is related to the dysfunction of neurotransmitters such as noradrenalin, dopamine and serotonin and changes in monoaminergic pathways [6]. It has also been hypothesised that depression and diabetes share a common neurobiological background associated with changes in brain white matter and impaired neuroplasticity [7].

The pathogenesis of depression could be linked with that of diabetes and its comorbidities, such as obesity, dyslipidaemia and hypertension, by low-grade chronic systemic inflammation. Chronic hyperglycaemia is associated with increased pro-inflammatory cytokines, elevated synthesis of advanced glycation end products (AGEs), and overproduction of reactive oxygen species (ROS). Furthermore, elevated oxidative stress, inflammatory responses, and disturbed blood–brain barrier permeability increases the leakage of proteins and other mediators into the perivascular space. This can allow various cytokines, such as IL-6, tumour necrosis factor (TNF-α), and IL-1β, to influence neurotransmitter metabolism, impair neuroendocrine function or neuroplasticity, and decrease neurogenesis, which plays a crucial role in the pathogenesis of depression [8,9,10,11]. Also, a body of research suggests that depression arises from neuroinflammation resulting from the stimulation of microglia or astrocytes and the secretion of pro-inflammatory cytokines. Neuroinflammation leads to structural and functional changes in brain and HPA activation [12,13].

It has also been hypothesised that seniors may be more at risk of mood disorders as a result of the ageing process. Ageing is believed to transition the body into a pro-inflammatory state, which is driven by raised immune reactions at a peripheral level, disrupted immune signalling between the central and peripheral nervous systems, and the central nervous system providing more intense and inconsistent responses [14]. However, the evidence regarding a relationship between inflammatory mediators and late-life depression is contradictory [15], and little concrete data exists concerning these issues in the oldest-old population with diabetes. This population could also be subject to certain atypical vascular and lifestyle factors, and the predictive model of depression becomes more complex in late life. Therefore, the aims of this study were to compare the levels of inflammatory markers (TNF-α, IL-1β, and IL-6) between oldest-old patients with diabetes with and without depressive symptoms and to identify the risk factors associated with depressive symptoms in this group.

## 2. Results

### 2.1. Demographic and Clinical Profile of Patients with T2DM

The demographic and clinical profiles of the study group are presented in Table 1 and Table 2. Subjects with depressive symptoms were more likely to be female, living alone and smoking, with no physical activity; in addition, they were more likely to be diagnosed with cardiovascular disease, retinopathy, neuropathy, hypertension and hyperlipidaemia, and a higher proportion was treated with insulin (χ^2^ test; Table 1). No significant differences between depressive and non-depressive symptom groups were found with regard to the presence of stroke, nephropathy, prevalence of mild cognitive impairment, or treatment with OAD. Also, patients with depressive symptoms tended to be older, with a longer duration of diabetes, an increased number of comorbidities, and higher BMI and fasting blood glucose; they also tended to present elevated levels of HbA_1c_, total cholesterol, LDL cholesterol and triglycerides, lower concentrations of HDL cholesterol, and higher GDS scores (t test or Mann–Whitney U test; Table 2). Lastly, no significant differences were found between the two groups with regard to years of education, MoCA score, or systolic or diastolic blood pressure.

### 2.2. Inflammatory Marker Levels in Patients with Depression and Controls

**Figure 1:**
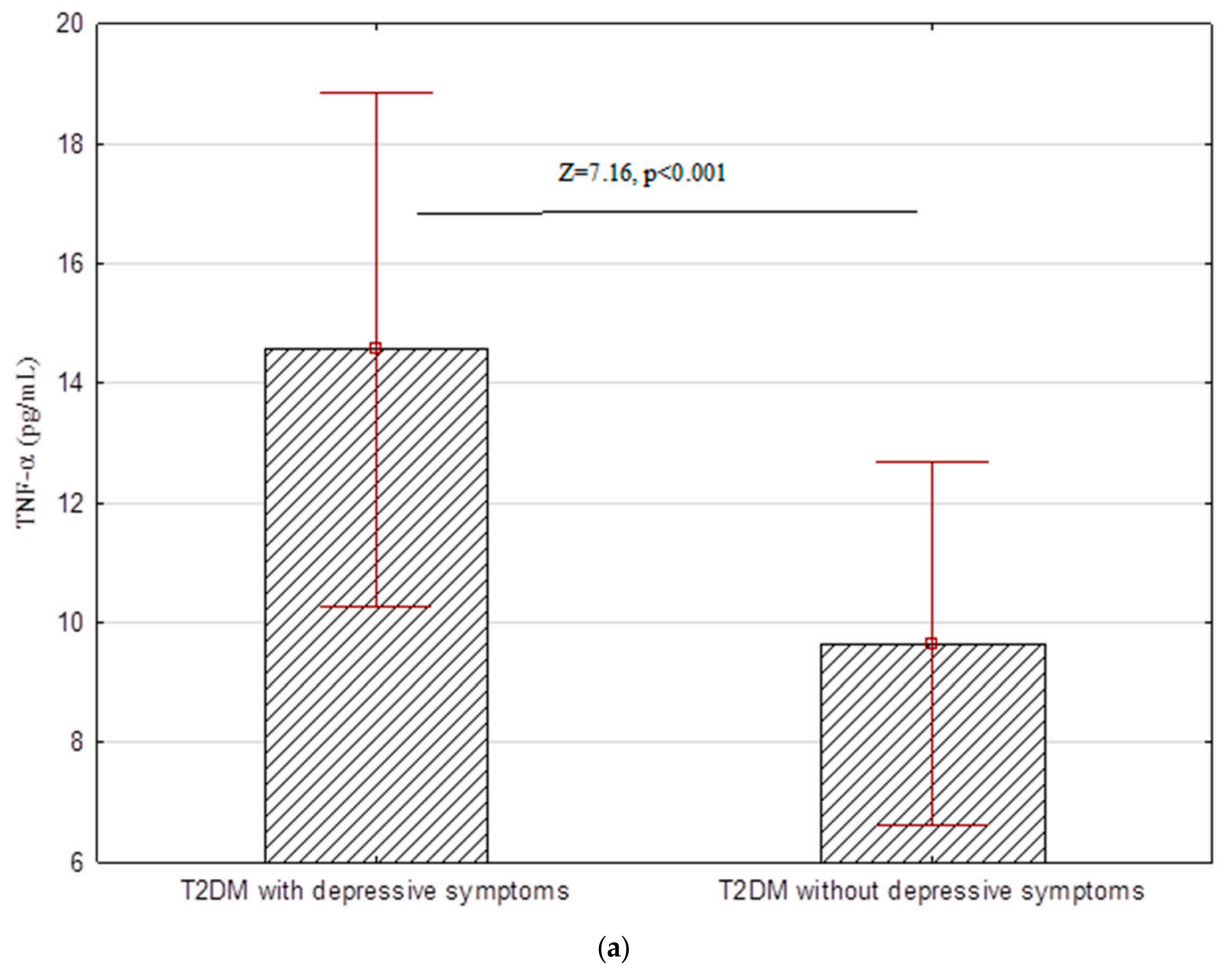

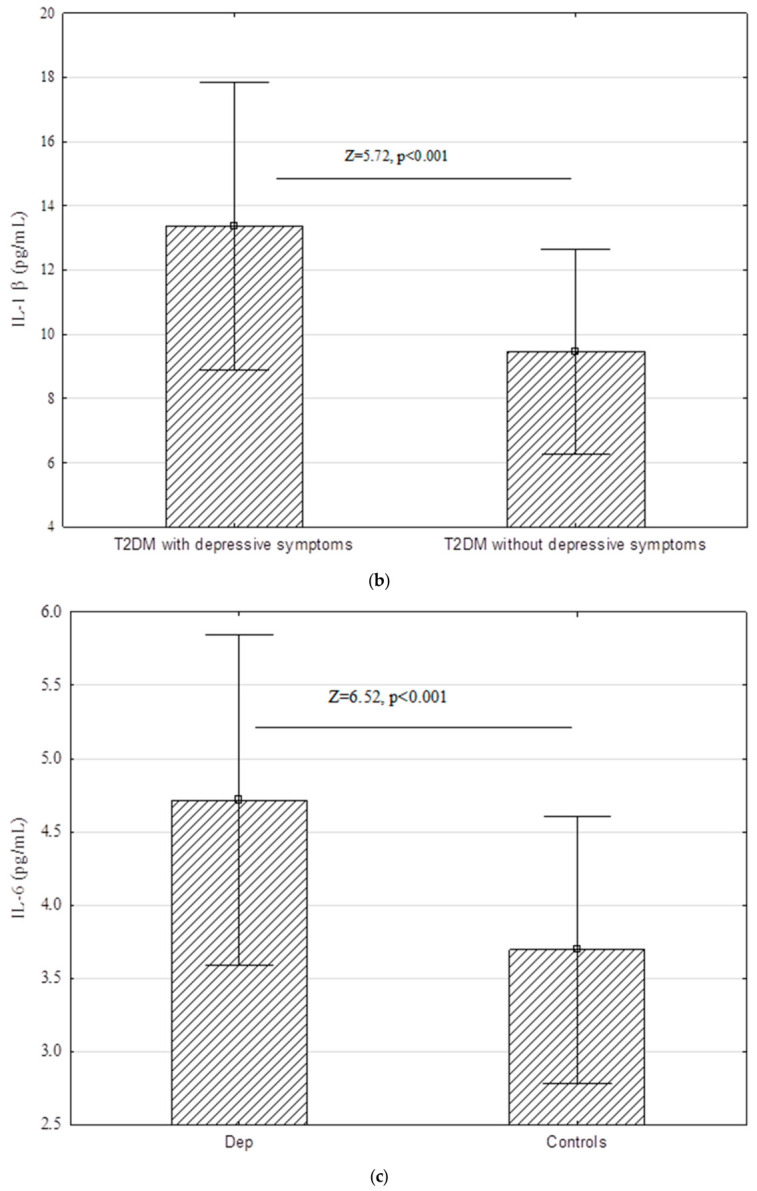
Serum levels of TNF-α (pg/mL) (**a**), IL-1β (pg/mL) (**b**), and IL-6 (pg/mL) (**c**) in oldest-old patients with diabetes with and without depressive symptoms. Data are expressed as mean ± SD.

The group of patients with depressive symptoms was characterised by significantly higher serum levels of TNF-α IL-1β, and IL-6 than the group without depressive symptoms (Figure 1a–c). More precisely, the measured TNF-α level was 14.59 ± 4.27 pg/mL compared to 9.69 ± 3.03 pg/mL in the group without depressive symptoms; IL-1β was 13.38 ± 4.47 pg/mL compared to 9.5 ± 3.2 pg/mL in the group without depressive symptoms; IL-6 was 4.72 ± 1.13 pg/mL compared to 3.71 ± 0.91 pg/mL in the group without depressive symptoms. When considering both groups, i.e., all subjects, the mean TNF-α level was 11.45 ± 4.24 pg/mL, IL-1β was 10.88 ± 4.15 pg/mL, and IL-6 was 4.07 ± 1.11 pg/mL.

### 2.3. Correlation Analyses

Correlation analysis confirmed a significant positive relationship between the levels of all inflammatory markers in patients with T2DM with depressive symptoms (Table 3). Furthermore, the concentrations of TNF-α, IL-1β, and IL-6 were highly correlated with HbA_1c_ levels, GDS scores, FBG and BMI. Also, a weak positive association was noted between TNF-α or IL-6 levels and the duration of diabetes and between TNF-α or IL-1β levels and triglyceride levels or the number of comorbidities, and finally, a weak negative relationship was noted between TNF-α or IL-1β levels and HDL cholesterol levels (Table 3).

Interestingly, in the group of patients with depressive symptoms, elevated HbA_1c_ levels were associated with higher GDS scores (r = 0.72, *p* < 0.001), BMI (r = 0.47 *p* < 0.001), and number of comorbidities (r = 0.65, *p* < 0.001). Also, higher GDS scores correlated with BMI (r = 0.63 *p* < 0.001) and the number of comorbidities (r = 0.64, *p* < 0.001) (data not presented in tables).

### 2.4. Risk Factor for Depressive Symptoms

According to the univariate logistic regression models, the following variables are associated with depressive symptoms in oldest-old patients with type 2 diabetes: an older age; female sex; living alone; a smoking habit; no physical activity; a higher BMI; longer duration of T2DM; an increased number of comorbidities; the presence of cardiovascular disease, retinopathy, neuropathy, hypertension and hyperlipidaemia; insulin treatment; higher levels of HbA_1c_, total cholesterol, LDL and TG; lower concentrations of HDL; and higher levels of TNF-α, IL-1β, and IL-6 (Table 4). Multivariable logistic regression analyses revealed distinct patterns across the three constructed models (Table 4). In Model 1 (demographic adjustment), higher levels of TNF-α remained a strong factor associated with depressive symptoms alongside older age. In Model 2 (metabolic adjustment), longer diabetes duration and elevated HbA1c levels emerged as dominant independent factors. In Model 3 (cardiovascular and co-morbidity adjustment), higher levels of TNF-α demonstrated independent significance alongside the increased number of comorbidities.

## 3. Discussion

The studied elderly patients with T2DM and depressive symptoms demonstrated significantly elevated levels of inflammatory mediators such as TNF-α, IL-1β, and IL-6 compared to subjects without depressive symptoms. Furthermore, univariate analysis confirmed that TNF-α, IL-1β, and IL-6 are associated with depressive symptoms in the tested oldest-old population with diabetes.

These results are consistent with those of previous studies indicating systemic inflammation as a mechanism linking depression and diabetes [10,16,17]. A large meta-analysis found markers such as TNF-α, C-reactive protein (CRP), and IL-6 to be elevated in subjects with depression compared to those without [18]. It has also been confirmed that ageing is associated with an increase in the levels of cytokines, particularly IL-6 and TNF-α, even in healthy individuals; this could be partly explained by the rise in adiposity and the reduction in sex hormone levels following menopause and andropause [19]. One longitudinal study confirmed associations between depression and IL-6 levels [20]; an elevated IL-6 level is believed to play a role in vascular damage by enhancing oxidative stress, impairing endothelial function and facilitating the progression of atherosclerosis [21]. In addition, elevated TNF-α is associated with a greater prevalence of atherosclerosis [22], and IL-6, TNF-α and CRP are independent predictors of cardiovascular events in older adults [19]. Also, increased secretion of pro-inflammatory cytokines such as TNF-α, IL-1β, IL-6 and adipokines has been implicated in endothelial dysfunction induced by hyperglycaemia, oxidative stress and AGE overproduction. This mechanism is believed to link diabetes with its complications and the development of atherosclerosis [23].

A number of human population studies confirm that TNF-α plays an essential part in IL-6 production and that its levels correlate with IL-6 concentrations [19]. This is in line with our present findings, which confirm a significant correlation between all studied pro-inflammatory parameters. In addition, increased levels of TNF-α, IL-1β, and IL-6 were found to be related to higher GDS scores, indicating that the severity of inflammation corresponds to more severe depressive symptoms. Indeed, previous studies in older populations report that increased IL-1β or CRP [24], IL-6 [25] or TNF-α levels [26] were significantly related to depression severity. One large analysis indicates that inflammatory parameters, especially IL-6 and TNF-α, may represent biomarkers for the development of late-life depression [27].

A significant finding of the present study is that patients with both depressive symptoms and T2DM tended to demonstrate higher levels of TNF-α, IL-1β, and IL-6. In addition, pro-inflammatory marker concentrations demonstrated significant correlations with other tested parameters, such as FBG or HbA_1c_ levels, in the subjects with depressive symptoms. Also, elevated HbA_1c_ levels were associated with higher GDS scores, BMI and number of comorbidities. These observations are aligned with the literature suggesting a potential bidirectional link between depression and diabetes. This is supported by a recent systemic review and meta-analysis of longitudinal large-scale studies, which concludes that increased HbA_1c_ levels are associated with a higher risk of depression and vice versa [28]. While our cross-sectional design prevents us from establishing the cause of bidirectional pathways, our multivariable analysis confirms that HbA_1c_ remains a significant factor associated with depressive symptoms. In the literature, several biological mechanisms have been hypothesised to account for the relationship between hyperglycaemia and the development of depression. For instance, experimental studies suggest that during chronic hyperglycaemia, plasma glucose can easily cross the blood–brain barrier, potentially contributing to oxidative stress in neurons, leading to brain atrophy via neural apoptosis [28]. Alternatively, low-grade inflammation, decreased brain-derived neurotrophic factor and vascular pathology may be induced in areas of the brain connected to mood [28]. Regarding all the abovementioned mechanistic interpretations that may connect inflammation, diabetes and depression, we strongly emphasise that these pathways were not assessed in this study and should be described more clearly as theoretical mechanisms rather than explanations supported by the existing data. Our present findings indicate that, inter alia, previous CVD, hypertension, hyperlipidaemia, microvascular complications, and higher levels of TNF-α, IL-1β, or IL-6, and poorer glycaemic control (higher HbA1c levels) increased the likelihood of depressive symptoms. Furthermore, participants with both depressive symptoms and diabetes exhibited elevated levels of inflammatory markers, which may be associated with vascular diseases; as a result, inflammation may occur after vascular incidents or even trigger or worsen them. Clearly, inflammation plays a manifold role in the relationship between depression, diabetes and vascular diseases. This is supported in large epidemiological studies, which have reported an association between depression and vascular pathology [29,30] or a greater prevalence of micro and macro complications in patients with diabetes [31], as well as higher morbidity [32]. Our multivariable analysis confirmed that of the three studied biomarkers, only TNF-α is independently associated with depressive symptoms when adjusting for demographic factors (Model 1) and cardiovascular burden (Model 3). However, its significance disappeared when controlling for glycaemic parameters (Model 2). This crucial finding indicates that the inflammatory effect of TNF-α is potentially mediated by the duration and metabolic severity of diabetes. Importantly, the association of TNF-α with depressive symptoms in patients with diabetes underscores the broader systemic reach of diabetic vascular complications. In this context, diabetes-related inflammatory signalling may also promote autonomic remodelling and cardiac sympathetic overactivation. This provides an additional pathway through which poor glycaemic control increases cardiovascular vulnerability, as recently demonstrated by Singh et al. [33]. Conversely, IL-1β and IL-6 lost their significance in all adjusted models, suggesting that their univariate associations were confounded by shared clinical and metabolic factors. These results collectively underscore the complex interplay between systemic inflammation, autonomic regulation, and metabolic control in the pathogenesis of diabetes-related complications.

Among patients with diabetes, the onset of depressive symptoms might also be associated with the psychological burden connected to difficulties in self-management, fear of complications, unhealthy lifestyle, loneliness and chronic stress [34,35]. Our present findings indicate the risk of depressive symptoms to be associated with older age, an increased number of comorbidities, longer duration of diabetes, female sex, living alone, smoking, lack of physical activity, higher BMI and insulin treatment; all these parameters are widely described in the literature [36,37,38]. Female sex is a known risk factor for depression and anxiety. Single relationship status has been found to promote loneliness and poorer mood and is more common in the older population. Also, while most studies report older age as a risk factor for depression, data obtained from a cohort with diabetes yielded contradictory results [39]. Interestingly, one large report indicates that while older individuals are less likely to experience severe depression, they are more than ten times more likely to demonstrate milder symptoms, such as minor depression, than the younger population [40]. Our analysis identified older age as a significant independent factor associated with depressive symptoms. However, interpreting these scores in patients aged 85 and above requires caution due to potential somatic confounding. In our study, the risk was minimised by using the 2006 European Alzheimer’s Disease Consortium standard. These criteria require preserved functional independence. Because participants maintained functional autonomy and MoCA scores were similar between groups, these results likely reflect genuine mood disturbances rather than physical decline.

Older patients typically report a longer duration of diabetes, with a greater number of complications and comorbidities, as well as a more complex treatment regimen (e.g., insulin). These factors directly increase disease burden and may contribute to psychological distress and a poorer quality of life.

Our findings indicate that patients with depressive symptoms were less physically active and demonstrated higher BMI. This is in line with a previous large meta-analysis, which confirmed a longitudinal bidirectional association between obesity and depression [41]. Our data also indicate that BMI is related to the levels of all three tested inflammatory markers, as well as with GDS scores and HbA_1c_ concentrations. We therefore hypothesise that the mechanism underlying the pathologic circle of coexisting obesity, poor glycaemic control and more severe depressive symptoms could be based around systemic inflammation.

However, studies on the interaction between cytokine levels, obesity and depression provide contradictory results [42,43,44]. While IL-6 levels were found to be elevated in subjects with obesity and depression [42,43], no such correlation was observed between TNF-α and obesity [44]. In the present study, a positive correlation was observed between triglyceride levels and TNF-α or IL-1β concentrations and negative associations between these mediators and HDL levels. Hyperlipidaemia was also identified as a factor associated with depressive symptoms in elderly patients with diabetes in univariate analysis. These observations imply a complex relationship that encompasses, among other factors, inflammatory markers, dysregulation of adipose tissue in obesity, dyslipidaemia, insulin resistance and beta cell dysfunction [45].

Our study provides a significant contribution to the understanding of oldest-old patients with diabetes, either with or without depressive symptoms. Among this group, those with symptoms of depression presented significantly increased serum levels of TNF-α, IL-1β and IL-6. Furthermore, all these inflammatory markers demonstrated significant correlations with HbA_1c_ levels, GDS scores or BMI in patients with depressive symptoms. The levels of TNF-α, IL-1β, and IL-6, HbA_1c_ or GDS scores were also related to the number of comorbidities. Finally, univariate and then multivariate analyses were used to identify the key risk factors associated with depressive symptoms in this population.

This research is not without its limitations. As the study used a cross-sectional approach, it is not possible to establish causal relationships or bidirectional pathways between glycaemic control, inflammation, and depressive symptoms. Longitudinal studies are required to confirm the chronological order of these associations. Also, the study was based on a single centre, which might restrict the applicability of the findings to other groups. Lastly, because of the limited group size and relatively high number of variables, the multivariable models could not simultaneously adjust for all potentially relevant confounding factors. To avoid statistical overfitting, we constructed separate, parsimonious models focusing on the most critical clinical and metabolic covariates.

## 4. Conclusions

Our finding indicates a significant link between systemic inflammation, particularly elevated TNF-α, and depressive symptoms in oldest-old patients with T2DM. In this group, the following parameters are associated with depressive symptoms in the univariate analysis: older age; female sex; living alone; a smoking habit; no physical activity; higher BMI; longer duration of T2DM; an increased number of comorbidities; the presence of cardiovascular disease, retinopathy, neuropathy, hypertension or hyperlipidaemia; insulin treatment; higher levels of HbA_1c_, total cholesterol, LDL and TG; lower concentrations of HDL; and higher levels of TNF-α, IL-1β, and IL-6.

In conclusion, TNF-α, rather than IL-1β and IL-6, appears to maintain a distinct association with depressive symptoms in oldest-old patients. This relationship is tightly dependent on glycaemic control. Further longitudinal cohort studies and randomised controlled trials are needed to establish the prospective nature of these links and evaluate specific inflammatory markers as potential therapeutic targets.

## 5. Materials and Methods

### 5.1. Participant Selection and Setting

A cross-sectional study was performed of individuals with T2DM aged 85 and above. All subjects were consecutively recruited from the outpatient diabetology clinic affiliated with University Teaching Hospital No 1 in Lodz, Poland. Written informed consent was obtained from the participants at the beginning of the study.

The inclusion criteria were as follows: age 85 and over, diagnosis of T2DM a minimum of one year earlier, full ability to understand and cooperate with study procedures and provision of written informed consent. The exclusion criteria included the following: pre-existing psychiatric disorders (e.g., bipolar disorder, schizophrenia or dementia); use of possible drugs with known psychotropic effects; active neoplasm or infectious disease; constant alcohol or substance abuse; severe visual, hearing, or motor coordination impairment; history of brain injuries or inflammatory or infectious brain disease.

### 5.2. Data Collection and Procedures

General demographic information was collected: sex, age, and years of education and living alone (single/widowed/divorced). A detailed medical history was obtained from each patient via interview. This included smoking habits, any physical activity, diabetes duration, the number of comorbid diseases, micro and macro complications of diabetes (hypertension, cardiovascular diseases, hyperlipidaemia, stroke, retinopathy, neuropathy, nephropathy), and current treatment for diabetes (oral or insulin). Body mass index (BMI) was determined by measuring height and weight and using the following formula: kg/m^2^. Systolic and diastolic blood pressure (mmHg) were taken with the patient seated after resting for 10 min. The diagnosis of hypertension was made based on either a past history of hypertension or the intake of any antihypertensive medications. Hyperlipidaemia was diagnosed based on either the use of any lipid lowering drug or the presence of untreated triglyceride levels of 1.7 mmol/L or serum LDL cholesterol of 2.6 mmol/L or higher.

Blood samples were collected though a blood draw in the morning following 10- to 12-h overnight fasting. The participants received a snack afterward to confirm they were not experiencing hypoglycaemia during the neuropsychological assessment. The participants then moved to a different, more peaceful room, where they gave a detailed medical history and received the full physical examination and psychological test battery.

### 5.3. Neuropsychological Assessment

Depressive symptoms were evaluated using the 30-item Geriatric Depression Scale (GDS) [46]. Scores ranging from 0 to 9 are considered non-depressive, while those between 10 and 19 indicate the presence of depressive symptoms. A score of 20 or more is classified as severe depression, and individuals with such scores were referred to a psychiatrist for additional evaluation.

Mild cognitive impairment (MCI) was evaluated using the Montreal Cognitive Assessment (MoCA) [47], which is known as the most effective screening instrument for MCI in elderly patients with diabetes [48]. The MoCA test comprises eight areas: visuospatial/executive reasoning, memory, naming, abstraction attention, language, and orientation skills. The highest possible score is 30 points, and a result of ≥26 is evaluated as cognitively normal. An additional point is added for those who have less than 12 years of formal education. For this research, the diagnosis of MCI was established based on criteria established by the 2006 European Alzheimer’s Disease Consortium [49]. These guidelines include absence of dementia. The diagnosis of MCI required both subjective cognitive complaints reported by the subject during the interview and preserved daily functioning (in our study, measured by Katz Basic Activities (BADL) and Lawton Instrumental Activities of Daily Living (IADL)). As indicated by findings from epidemiological studies, MoCA scores under 19 points were interpreted as indicative of dementia; these subjects were excluded from the study and referred to a psychiatrist for further assistance.

### 5.4. Biochemical Analyses

Blood samples were collected, centrifuged and stored in separate aliquots at −80 °C for a maximum of 6 months to avoid freeze–thaw cycles and ensure cytokine stability; all assays were run in duplicate. The levels of TNF-α (Human TNF-α; DTA00C), IL-1β (Human IL-1β; DLB50), and IL-6 (Human IL-6; D6050) were determined by the Quantikine Human Immunoassay ELISA kit (Bio-techne, Minneapolis, MN, USA) according to the manufacturer’s instructions. For TNF-α, the intra-assay coefficient of variation was 5.2% and the inter-assay coefficient was 7.4%. For IL-1β, the intra-assay coefficient was 8.5% and the inter-assay coefficient was 8.4%. For IL-6, the intra-assay coefficient was 1.8% and the inter-assay coefficient was 4.2%. The minimum detectable concentrations were 1 pg/mL for IL-1β, 1.6 pg/mL for TNF-α, and 0.626 pg/mL for IL-6.

Glycosylated haemoglobin (HbA_1c_), fasting blood glucose (FBG), total cholesterol, triglycerides, low-density lipoprotein cholesterol (LDL) and high-density lipoprotein cholesterol (HDL) were measured in a centralised laboratory.

### 5.5. Group Selection

The 195 participants with T2DM recruited to the study were divided into two groups: 70 (35.9%) patients with depressive symptoms and 125 (64.1%) subjects without depressive symptoms. The second group acted as controls.

### 5.6. Ethics

The study was carried out in full compliance with the guidelines given in the Helsinki Declaration. Every patient received a specific identification number to maintain confidentiality. The purpose, nature, and potential risks of the experiments were fully explained to the participants. All participating patients provided their written informed consent to take part at the start of the study. Ethical approval was obtained from the independent local ethics committee of the Medical University of Lodz (Approval No. RNN/420/13/KB).

### 5.7. Statistical Analysis

The statistical analysis was performed using Statistica 13.1 (StatSoft, Krakow, Poland). Categorical variables were expressed as numbers and percentages, whereas continuous variables were reported as means with standard deviations (SDs). Normality was determined by the Shapiro–Wilk test. Among the continuous variables, normally distributed ones were compared using the independent *t* test and non-normally distributed ones with the Mann–Whitney U test. Categorical data were analysed using the chi-square (χ^2^) test. The relationships were assessed using Pearson’s correlation analysis for normally distributed variables or Spearman’s rank correlation for non-normally distributed ones. A simple logistic regression model was developed to identify the independent factors that increase the selection risk of depressive symptoms in elderly patients with T2DM. To evaluate the independent associations of the studied inflammatory mediators with depressive symptoms, multivariable logistic regression analyses were performed using the standard Enter method. To prevent model overfitting and adhere to the events-per-variable guidelines, three separate parsimonious models were constructed: Model 1 adjusted for basic demographic factors, including age and sex; Model 2 adjusted for metabolic parameters such as diabetes duration and HbA_1c_ levels; and Model 3 adjusted for CVD and the number of comorbid diseases. Odds ratios (ORs) were computed and presented with the 95% interval of confidence (CI). *p*-values of < 0.05 were considered statistically significant.

## Figures and Tables

**Table 1:** Baseline demographic and clinical characteristics by study group.

Characteristic	All Patients	T2DM with Depressive Symptoms	T2DM Without Depressive Symptoms (Comparison Group)	χ^2^	*p* Value
Number of patients	195	70 (35.9%)	125 (64.1%)		
Sex, female *	107 (54.8%)	49 (70%)	58 (46.4%)	10.1	0.0015
Living alone *	90 (46.2%)	47 (67.14%)	43 (34.4%)	19.4	<0.001
Smoked tobacco regularly *	56 (28.7%)	27 (38.6%)	29 (23.2%)	5.18	0.02
No physical activity *	143 (73.2%)	64 (91.4%)	79 (63.2%)	17.7	<0.001
Macrovascular complications CVD*	124 (63.6%)	58 (82.6%)	66 (52.8%)	17.5	<0.001
Stroke	9 (4.6%)	5 (7.1%)	4 (3.2%)	1.58	0.21
Previous HA/use of HA drugs *	162 (83.1%)	64 (91.4%)	98 (78.4%)	4.99	0.025
Hyperlipidaemia*	164 (84.1%)	66 (94.3%)	98 (78.4%)	7.96	0.0048
Microvascular complications Retinopathy *	126 (64.6%)	60 (85.7%)	66 (52.8%)	21.26	<0.001
Nephropathy	95 (48.7%)	35 (50%)	60 (48%)	0.07	0.78
Neuropathy *	34 (17.4%)	19 (27.1%)	15 (12%)	7.42	0.006
MCI	90 (46.6%)	38 (54.3%)	52 (41.6%)	1.05	0.3
Treatment OAD	181 (92.8%)	64 (91.4%)	117 (93.6%)	0.61	0.43
Insulin *	77 (39.5%)	37 (52.8%)	40 (32%)	8.17	0.004

Abbreviations: T2DM—type 2 diabetes, MCI—mild cognitive impairment, CVD—cardiovascular disease, HA—hypertension, OAD—oral antidiabetic drugs. Values are presented as numbers (percentages); χ^2^ test was used to test for significant differences; * significance *p* < 0.05.

**Table 2:** Characteristics and biochemical parameters by study group.

	All Patients	T2DM with Depressive Symptoms	T2DM Without Depressive Symptoms (Comparison Group)	Z/t	*p* Value
Number of patients	195	70 (35.9%)	125 (64.1%)		
Age [years] *	87.3 ± 2.6	88.2 ± 2.6	86.8 ± 2.6	4.34	<0.001
Education-years	10.9 ± 2.7	10.7 ± 2.8	11.1 ± 2.6	−1.2	0.22
Duration of T2DM [years] *	20.1± 8	25.3 ± 7.2	17.2 ± 6.8	7.4	<0.001
BMI [kg/m^2^] *	29.7 ± 3.8	32.5 ± 2.9	28.1 ± 3.4	7.7	<0.001
HbA_1c_ [%] *	7.5 ± 0.9	8.19 ± 0.7	7.1 ± 0.7	8.34	<0.001
CHOL [mmol/L] *	4.94 ± 0.99	5.45 ± 0.94	4.66 ± 0.91	5.7	<0.001
LDL [mmol/L] *	2.88 ± 0.8	3.25 ± 0.81	2.67 ± 0.71	5.1	<0.001
TG [mmol/L] *	2.05 ± 0.4	2.16 ± 0.37	1.98 ± 0.41	3.1	0.002
HDL [mmol/L] *	1.12 ± 0.23	1.07 ± 0.21	1.15 ± 0.23	−2.5	0.01
Co-morbidity [n] *	6.2 ± 3.6	9.1 ± 2.7	4.6 ± 2.9	8.1	<0.001
GDS score *	7.9 ± 6.9	16.34 ± 2.5	3.1 ± 2.7	11.6	<0.001
MoCA score	24.6 ± 3.2	24.3 ± 3.3	24.7 ± 3.1	−0.8	0.4
Systolic blood pressure [mm Hg]	133.7 ± 15.6	132.8 ± 17.1	134.3 ± 14.7	−1.5	0.12
Diastolic blood pressure [mm Hg]	75.1 ± 7.5	74.6 ± 7.2	75.3 ± 7.6	−0.5	0.6
Fasting blood glucose [mmol/L] *	7.35 ± 1.32	7.6 ± 1.42	7.22 ± 1.25	2.1	0.03

Abbreviations: T2DM—type 2 diabetes, BMI—body mass index, HbA_1c_—glycosylated haemoglobin, CHOL—total cholesterol, LDL—low-density lipoprotein cholesterol, HDL—high-density lipoprotein cholesterol, TG—triglycerides, GDS—Geriatric Depression Scale, MoCA—Montreal Cognitive Assessment. Values are presented as mean ± standard deviations; Mann–Whitney U test (Z) or *t* test was used to test for significant differences, * significance *p* < 0.05.

**Table 3:** Quantitative relationship between serum levels of TNF-α (pg/mL), IL-1β (pg/mL), and IL-6 (pg/mL) on other selected parameters among elderly patients with diabetes with depressive symptoms.

Parameter	TNF-α (pg/mL)		IL-1β (pg/mL)		IL-6 (pg/mL)	
	r	*p*	r	*p*	r	*p*
Duration of T2DM [years]	0.25 *	0.03	-	-	0.44 *	<0.001
BMI [kg/m^2^]	0.62 *	<0.001	0.46 *	<0.001	0.47 *	<0.001
HbA_1c_ [%]	0.8*	<0.001	0.66 *	<0.001	0.49 *	<0.001
CHOL mmol/L)	-	-	-	-	-	-
LDL (mmol/L)	-	-	-	-	-	-
TG [mmol/L]	0.27 *	0.02	0.42 *	<0.001	-	-
HDL [mmol/L]	−0.28 *	0.02	−0.43	<0.001	-	-
Co-morbidity [n]	0.51 *	<0.001	0.43 *	<0.001	-	-
GDS score	0.69 *	<0.001	0.45 *	<0.001	0.56 *	<0.001
Fasting blood glucose [mmol/L]	0.5*	<0.001	0.38 *	0.001	0.28 *	0.02
TNF-α (pg/mL)	-	-	0.79 *	<0.001	0.61 *	<0.001
IL-1β (pg/mL)	0.79 *	<0.001	-		0.47 *	<0.001
IL-6 (pg/mL)	0.61 *	<0.001	0.47*	<0.001	-	-

* statistically significant difference, *p* < 0.05; r-correlation coefficient. Abbreviations: T2DM—type 2 diabetes, BMI—body mass index, HbA_1c_—glycosylated haemoglobin, CHOL—total cholesterol, LDL—low-density lipoprotein cholesterol, HDL—high-density lipoprotein cholesterol, TG—triglycerides, GDS—Geriatric Depression Scale.

**Table 4:** Univariate and multivariable logistic regression analysis of factors associated with depressive symptoms in oldest-old patients with diabetes.

Parameter	Univariate Analysis OR (95% CI); *p*-Value	Model 1 (Demographic) OR (95% CI); *p*-Value	Model 2 (Metabolic) OR (95% CI); *p*-Value	Model 3 (CVD/Co-Morbidity) OR (95% CI); *p*-Value
TNF-α [pg/mL]	1.38 (1.2–1.5); *p* < 0.001 *	1.41 (1.18–1.68); *p* < 0.001 *	1.07 (0.85–1.35); *p* = 0.561	1.27 (1.04–1.53); *p* = 0.017 *
IL-1β [pg/mL]	1.28 (1.17–1.39); *p* < 0.001 *	0.95 (0.81–1.11); *p* = 0.518	0.95 (0.81–1.12); *p* = 0.558	0.93 (0.78–1.19); *p* = 0.366
IL-6 [pg/mL]	2.69 (1.8–3.8); *p* < 0.001 *	1.09 (0.66–1.81); *p* = 0.729	0.95 (0.56–1.60); *p* = 0.842	1.17 (0.67–2.03); *p* = 0.585
Age [years]	1.24 (1.09–1.39); *p* < 0.001 *	1.31 (1.13–1.51); *p* < 0.001 *	-	-
Sex, female	2.69 (1.45–5.01); *p* = 0.002 *	1.88 (0.85–4.18); *p* = 0.121	-	-
Duration of T2DM (years)	1.16 (1.1–1.2); *p* < 0.001 *	-	1.10 (1.04–1.16); *p* < 0.001 *	-
HbA_1c_ (%)	6.9 (4.1–11.7); *p* < 0.001 *	-	4.84 (1.92–12.19); *p* = 0.001 *	-
CVD	4.32 (2.1–8.8); *p* < 0.001 *	-	-	1.09 (0.40–2.99); *p* = 0.860
Co-morbidity [n]	1.6 (1.4–1.8); *p* < 0.001 *	-		1.40 (1.21–1.63); *p* < 0.001 *
Education-years	0.9 (0.84–1.04); *p* = 0.26	-	-	-
BMI [kg/m^2^]	1.46 (1.3–1.6); *p* < 0.001 *	-	-	-
CHOL [mmol/L]	1.02 (1.01–1.03); *p* < 0.001 *	-	-	-
LDL [mmol/L]	1.03 (1.01–1.04); *p* < 0.001 *	-	-	-
TG [mmol/L]	1,01 (1.004–1.03); *p* = 0.005 *	-	-	-
HDL [mmol/L]	0.96 (0.92–0.99); *p* = 0.01 *	-	-	-
MoCA score	0.9 (0.8–1.1); *p* = 0.38	-	-	-
Systolic blood pressure [mm Hg]	0.9 (0.97–1.01); *p* = 0.9	-	-	-
Diastolic blood pressure [mm Hg]	0.98 (0.95–1.03); *p* = 0.56	-	-	-
Fasting blood glucose [mmol/L]	1.03 (1.00–1.03); *p* = 0.052	-	-	-
Living alone	3.8 (2.09–7.25); *p* < 0.001 *	-	-	-
Smoked tobacco regularly	2.1 (1.1–3.9), *p* = 0.02 *	-	-	-
No physical activity	6.0 (2.4–14.9); *p* < 0.001 *	-	-	-
Stroke	2.3 (0.6–8.9); *p* = 0.21	-	-	-
Previous HA/use of HA drugs	2.8 (1.09–7.18)’ *p* = 0.03 *	-	-	-
Hyperlipidaemia	4.3 (1.4–12.9); *p* = 0.008 *	-	-	-
Retinopathy	5.36 (2.5–11.4); *p* < 0.001 *	-	-	-
Nephropathy	1.08 (0.6–1.90; *p* = 0.78	-	-	-
Neuropathy	2.9 (1.38–6.2); *p* = 0.005 *	-	-	-
MCI	1.6 (0.9–3.0); *p* = 0.08	-	-	-
Treatment OAD	0.6 (0.2–1.9); *p* = 0.42	-	-	-
Insulin	2.29 (1.2–4.18); *p* < 0.001 *	-	-	-

* Statistically significant difference, *p* < 0.05; CI: confidence interval for odds ratio; OR: odds ratio; Model 1 adjusted for age and sex; Model 2 adjusted for duration of T2DM and HbA_1c_; Model 3 adjusted for CVD and number of comorbidities. Abbreviations: T2DM—type 2 diabetes, MCI—mild cognitive impairment, BMI—body mass index, HbA_1c_—glycosylated haemoglobin, CHOL—total cholesterol, LDL—low-density lipoprotein cholesterol, HDL—high-density lipoprotein cholesterol, TG—triglycerides, GDS—Geriatric Depression Scale, MCI—mild cognitive impairment, CVD—cardiovascular disease, HA—hypertension, OAD—oral antidiabetic drugs.

## Data Availability

The data presented in this study are available on request from the corresponding author.
